# Effect of aquatic versus conventional physical therapy program on ankle sprain grade III in elite athletes: randomized controlled trial

**DOI:** 10.1186/s13018-024-04855-0

**Published:** 2024-07-11

**Authors:** Maryam M. Sadaak, Salwa Fadl AbdElMageed, Mona Mohamed Ibrahim

**Affiliations:** 1 Smouha Sporting Club, Alexandria, Egypt; 2 Department of Physical Therapy for Musculoskeletal Disorders and its Surgeries, Faculty of Physical Therapy, Cairo University, Cairo, Egypt

**Keywords:** Conventional physical therapy, Sports injuries, Ankle sprain, Hydrotherapy, Aquatic, Grade III

## Abstract

**Introduction:**

Ankle sprains are the second most common sports injury after knee injuries, with about 85% of them affecting the lateral ankle ligaments. These injuries are particularly prevalent in sports like basketball and volleyball.

**Purpose:**

To investigate the effect of Aquatic therapy as an early rehabilitation protocol for elite athletes with acute lateral ankle sprain grade III on back-to-sport time, dynamic balance, pain, Athletic performance, and muscle power compared to land-based exercise training.

**Methods:**

Thirty elite athletes have ankle sprain grade III with sprain onset from 1 to 7 days, their age ranges from 18–30 years old were recruited. All participants are professional athletes; mainly participating in above-head sports such as volleyball and basketball. The patients were randomly allocated into two treatment groups: Group I (control group): 15 patients received a conventional physical therapy program of structured therapeutic exercise program, manual therapy and land-based exercises, in addition to external support, and Group II (Aquatic therapy group): 15 patients received aquatic training. Visual Analog Scale (VAS) was used to measure the pain intensity, while the dynamic balance was measured by the Star Excursion Balance Test. Athletic performance was measured by HOP Tests (Single, Triple, 6-m, and Cross-over hops) aided by the Agility T-Test (ATT) and Illinois Agility Test (IAT). Muscle power was tested by a Single Leg Press. Finally, back to sports time was recorded for each participant in both groups.

**Results:**

There was a significant interaction effect of Aquatic therapy and time for VAS (*p* < 0.001), single hop (*p* < 0.001), triple hop (*p* < 0.001), cross-over hop (*p* < 0.001), IAT (*p* = 0.019) and ATT (*p* < 0.001) of both affected and nonaffected. There was no significant interaction effect of Aquatic therapy and time for 6-MHT of affected (*p* = 0.923), and nonaffected (*p* = 0.140). There was a significant main effect of time for all dependent variables (*p* < 0.001) except for 6-MHT of affected (*p* = 0.939), nonaffected (*p* = 0.109), and IAT (*p* = 0.099). The Star excursion dynamic balance test (SEBT) and Single leg press revealed a significant difference between groups on affected and non-affected sides (*p* < 0.001*). Lastly and most importantly the back-to-sport time revealed a significant difference in the return-to-sport time in favor of the Aquatic therapy group who returned faster than the control group (*p* < 0.001*).

**Conclusion:**

Aquatic therapy is more effective than traditional protocols regarding early rehabilitation of acute ankle sprain grade III in Elite professional athletes for reducing pain intensity, improving dynamic balance and athletic performance and power and accelerating their return to sports time. Because aquatic therapy produces better outcomes, it is advised to be included in the rehabilitation programs of athletic patients with acute ankle sprains grade III.

## Introduction

Ankle sprains are the second most common sports injury after knee injuries [35], with about 85% of them affecting the lateral ligaments due to inward twisting. this extremely high rate of injury was justified by multiple reasons including but not limited to jumping and landing mechanisms; because these actions increase the risk of missteps and awkward landings putting significant stress on the ankles and making them more suspectable to sprains. Also, rapid direction changes as these actions require great agility and proprioception thus, the shifts and pivoting manoeuvre increases the chances of falling or twisting the ankle. Moreover, landing on other players’ feet, uneven playing surfaces, intense training and a competitive environment were all adding reasons to that increase in injury rate [13]. These injuries are more prevalent in female athletes, particularly in high-risk sports like basketball and volleyball [15, 16, 35].

Ankle injuries account for 14% of orthopaedic emergency visits related to sports [22]. Approximately 50% of those who experience an ankle sprain don't seek medical attention, leading to over half of the players missing at least one week of competition [15]. Moreover, there is a high recurrence rate of about 70%, which can result in chronic ankle instability (CAI), leading to pain, limited physical activity, and an increased risk of osteoarthritis and degeneration of the talus joint [22]. Patients with acute ankle sprains typically present with localized pain, swelling, bruising, tenderness, loss of function, instability, and in some cases, paresthesia due to neurovascular compromise or peroneal nerve injury [33].

Physiotherapy, incorporating functional therapy along with a pain-free active range of motion [12], manual therapy, anteroposterior manipulation, therapeutic exercise [19], taping [27], muscle strength training, stability training, and sport-specific exercises, has shown promising results compared to immobilization and the PRICE (Protection, Rest, Ice, Compression and Elevation) protocol only during the inflammatory phase [34, 37].

The use of a semi-rigid cast in the early phase of ankle sprain was associated with faster recovery and higher satisfaction compared to elastic bandages [31]. In cases where conservative treatment fails to improve stability, pain, or function after 3–6 months surgical intervention with the modified Broström technique as the primary approach was recommended, followed by the anatomical reconstruction approach when there is a lack of adequate tissues for repair [10].

Aquatic therapy, which includes exercises underwater, is an excellent exercise medium for managing acute ankle sprains as the hydrostatic pressure of the water nullifies the gravity effects [30]. Aquatic therapy can decrease acute and chronic pain during weight bearing, induce faster recovery of the damaged ligaments, enhance static and dynamic stability, and percentage of single-limb support time of the affected leg according to recent studies [2, 25].

Aquatic therapy can help athletes with deficits secondary to ankle sprain by facilitating early rehabilitation through water buoyancy, reducing pressure over the joints, decreasing pain and swelling, and increasing strength, flexibility, and range of motion [25]. It also provides a low-impact environment for athletes, reducing stress on the joints and allowing them to safely perform exercises that may not be possible on land. Underwater exercises can improve balance and stability in athletes with ankle sprains by enhancing proprioception, leading to improved joint stability and better performance on land-based activities [32].

In 2008, according to Hubbard and Hicks-Little, aquatic training can benefit the sports population by reducing the issue of severe acute ankle sprains that affect the performance of the players, the morale of the team, and the competition-winning rate. Positive results of this study may reduce social and psychological issues in addition to musculoskeletal problems [24].

The psychological impact of this kind of injury on athletes is great affecting their self-esteem and developing a higher level of anxiety and depression [15]. Furthermore, returning to sports time is an important issue in athletic careers which affects their ranking and achievement in addition to the club's performance. Rapid return to the field without such good performance and power may be associated with higher rates of recurrent injuries often observed especially with lateral ankle sprain [39].

Up to the author’s knowledge, no previous studies are available to document the efficiency of Aquatic therapy and exercises on the recovery time of a severe acute ankle sprain while maintaining a higher level of proprioception and balance of the player. It shall be hypothesized that there will be a significant decrease in the back-to-sports time & pain intensity level while a significant increase in the score of star excursion balance test (SEBT), athletic performance, and muscle power in patients with ankle sprain grade III while using aquatic exercises in early rehabilitation compared to land-based rehabilitation.

This study will help the researchers interested in grade III ankle sprain early rehabilitation in providing the best treatment in the shortest back-to-sport time with excellent results, Therefore, this study aims to investigate the effect of a suggested Aquatic therapy protocol as an early rehabilitation intervention for elite athletes with acute lateral ankle sprain grade III on recovery time, dynamic balance, proprioception, pain, performance, and muscle power compared to land-based exercise training.

## Materials and methods

**Fig. 1:**
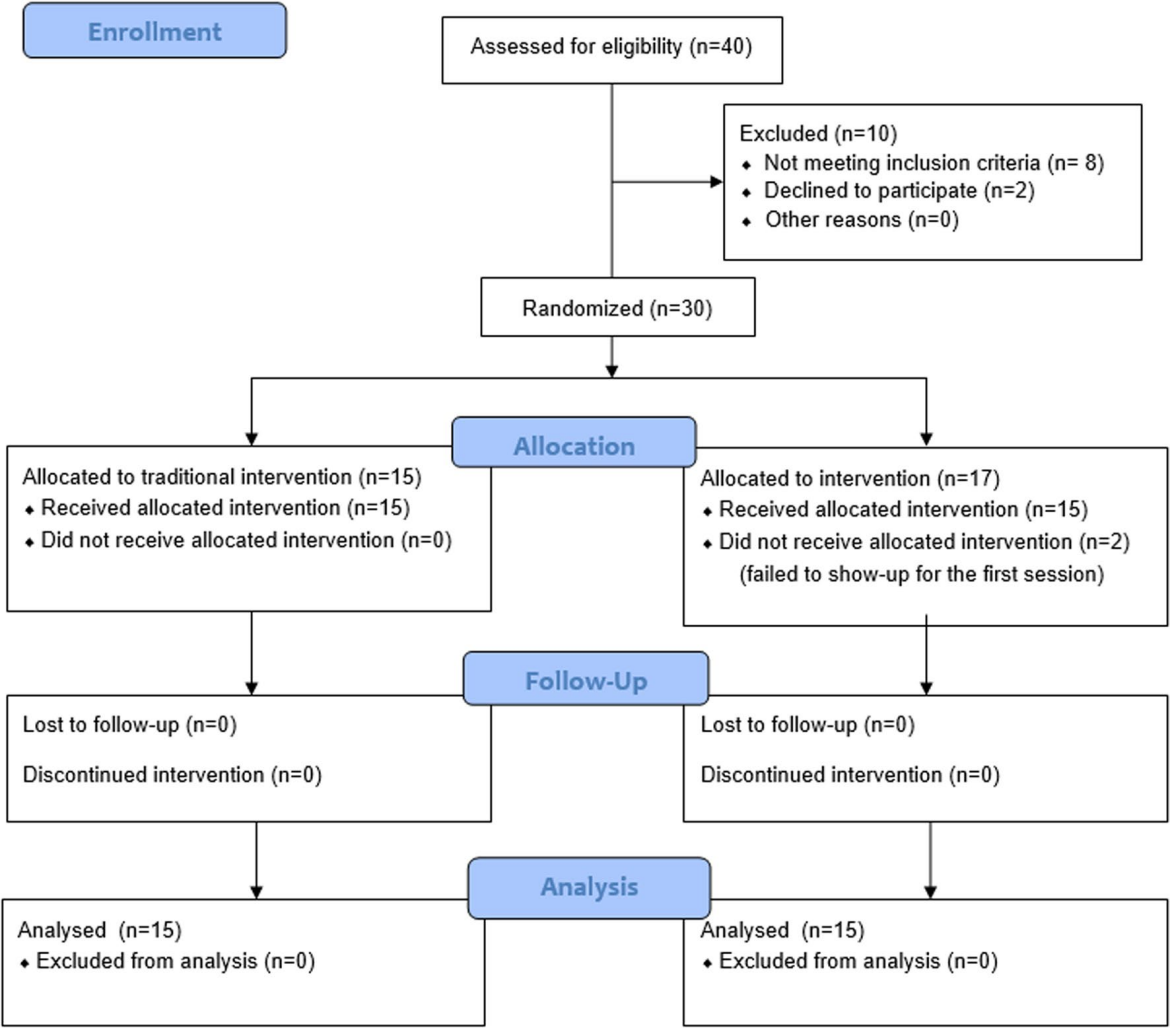
Flow chart of screened and included patients

A sample of 30 patients was recruited (Fig. 1), all participants are professional athletes; mainly participating in above-head sports such as Volley-ball and basketball and secondly participants of high contact sports such as Football and Rugby and have ankle sprain grade III with sprain onset from 1–7 days, their age ranges from 18–30 years old. The patients were randomly allocated into two treatment groups; Group I (control group): 15 patients received a structured therapeutic exercise program, manual therapy and land-based exercises, with the assistance of external support and Group II (Aquatic therapy group): 15 patients received complete protocol of underwater training. The suggested training protocol was extended for four weeks and the assessment of pain and function was performed three times immediately after injury and after application of the training protocol once at week 4 and once at week 6. While assessment of dynamic balance, athletic performance and muscle power was performed after training protocol once at week 4 and once at week 6. The return to sports time was measured in both groups after 4 weeks.

All participants were diagnosed with grade III ankle sprain according to MRI investigations [21]; both genders and ages ranged between 18–30 years and BMI ranged between 18–25; any patient who was found to have a fracture through the x-ray investigations was excluded. Clinical testing which was also included are positive anterior drawer test or positive inversion stress test, any patient with a positive squeeze test, external rotation test or fibular translation test was diagnosed as having syndesmosis (high ankle sprain) patients and was excluded from this study [36]. Any athlete indicated for surgery or casting, who had subacute symptoms with more than 7 days, with a high ankle sprain or Pott’s fracture, had a previous history of musculoskeletal injury to the lower extremity or contraindicated for aquatic therapy acc according to Australian Physiotherapy Association guidelines (2021) were all excluded [26].

### Treatment procedure

All groups were treated by the same physical therapist. Group I received a rehabilitation protocol approved by the Clinical Practice Guidelines Linked to the International Classification of Functioning, Disability and Health from the Academy of Orthopedic Physical Therapy of the American Physical Therapy Association [26], while group II received the aquatic therapy program [40].

Visual Analog Scale (VAS) was used to measure the pain intensity [18]. The level of pain ranges from none on the left side of the scale to extremely intolerable pain on the right side according to the patient’s perspective. The assessment of the dynamic balance was measured by the Star Excursion Balance Test (SEBT) in which participants were instructed to reach 8 times in the the eight directions of a star drawn on the ground while standing on the affected leg [29].

The functional performance was assessed by the HOP tests which are a set of tests used to assess an athlete's performance and ability to return to sports after injury according to the level of his functional performance tests [14]. These tests include the Single hop test (SHT), Triple hop test (THT), Cross-over hop test (CHT), and 6-m hop test. In the Single hop test, the patient was asked to stand on one leg jump as far as possible and land firmly without losing balance. Distance is measured from the start line to the tip of the toe of the landing leg in cm [5]. In the Triple hop test, the patient was asked to jump on a single leg as far as possible, three consecutive times without losing balance. The distance was measured from the start line to the tip of the toe of the landing leg in cm [6]. In the Cross-over hop test, the patient was asked to stand on one side of the midline, then jump forward as far as possible, then crossover to the other side of the midline, and jump for the second time as far as possible, then side jump again to the first side of the midline and jump for the third time as far as possible without losing balance or falling. The distance was measured from the start line to the tip of the toe of the landing leg in cm [3]. In the 6-m hop test, the patient was asked to jump as fast as possible on a single leg over a distance of 6 m without losing balance or falling while measuring the time using a stopwatch in seconds [4]

The Illinois Agility Test (IAT) [23] and the Agility T-test [8] were used to measure the athlete's ability to change direction, accelerate, decelerate, and move laterally. It involves forward, lateral, and backward movements which are used for proprioception and athletic performance level assessment**.** Lastly, the muscles of the lower limb strength levels were assessed by the single-leg press test [9].To return to sports, athletes need to be able to do 10 reps of the single leg press with 1.5 times their body weight Finally, back-to-sport time was recorded for each participant in both groups.

The control group received a conventional physical therapy rehabilitation program constructed by approved by the Clinical Practice Guidelines Linked to the International Classification of Functioning, Disability and Health from the Academy of Orthopedic Physical Therapy of the American Physical Therapy Association [26] which consisted of; through the first-week means of immobilization were made by bracing and external support [1], Low-LASER therapy was used for managing the pain [11], occupational training [26], lymphatic drainage [12], therapeutic exercises in the form of active ROM (ankle pumps), active assisted eversion/inversion, stretching exercises [7] and neuromuscular training through towel curls.

In the second week, resistive ankle ROM was maintained through resisted dorsiflexion/planter-flexion, resisted inversion/eversion, postural re-education by doing toe raises and heel walking, balance training by lunging on stable/un-stable surfaces, step-ups/downs, lateral step-ups/downs [7] and lastly manual therapy techniques: joint mobilization, talar mobilization and 1st metatarso-phalangeal joint mobilization grade I [37].

All techniques were done in relevance to the pain and the ability of the participants to perform them. In the third and fourth weeks, the exercise progressed to Mini squat on an unstable surface, Single-leg stance while playing catch, Single-leg stance while playing with the coach, Single-leg stance with lower limb movement and Single-leg stance with lower-limb movement on an unstable surface regarding the balance training. The manual therapy included deep friction massage, joint mobilization, talar mobilization and 1st metatarso-phalangeal joint mobilization grade II/III was included in the program.

The aquatic therapy exercise group received under-water training from the first week as follows: warm-up by doing Forward/backwards walking, Lateral walking, Lateral cross-over stepping, Straight-leg walking and deep-water bicycle, stretching exercises that included the posterior calf and tibialis anterior muscles [40].

The exercise protocol was progressed through the second and third weeks by doing mobility training that consisted of planter/dorsiflexion using modified resistance fin, strengthening exercise that included hip extension, double-leg squat, single-leg squat, forward lunge, hip abduction from standing and lateral step-ups using elastic bands and under-water weights [40].

In the fourth week the patient was instructed for the aquatic therapy protocol in which the proprioception training was applied by doing forward lunges on a step while using dumbbells, single leg stance while tossing the ball and squats on a modified under-water balance board, functional training was introduced by doing vertical jumping and stationary running using a resistance cord [40].

## Data analysis

Statistical analysis was conducted using SPSS for Windows, version 26 (SPSS, Inc., Chicago, IL). Before final analysis, data were screened for normality assumption, homogeneity of variance, and presence of extreme scores and the *p*-value was set at < 0.05. This analysis was done as a pre-requisite for parametric testing of the analysis of differences. A sample of convenience was used based on the availability of the professional athletes with the specified inclusion criteria. However, a statistical power analysis was performed after the end of the study using return to sport time and the power of the recruited sample (n = 30) reached 99%.

Comparison between mean values of the different parameters in the two groups was performed using repeated measure MANOVA test to determine the significant differences between both groups at the two times testing interval (after 4 and 6 weeks of intervention). Independent sample t-test was used for between-group comparisons and paired sample t-test was used for within-group comparisons.

## Results

The flow chart of the screened and included patients is illustrated in Fig. 1. Comparing the mean values of age, and BMI for all patients in the Control and Aquatic therapy groups using the independent sample *t*-test revealed that there were no significant differences between them in age (*p* = 0.920), and BMI (p = 0.399) (Table 1).

**Table 1:** Descriptive statistics and the independent sample t-test for the mean values of age, and BMI of all patients in the Control and Aquatic therapy groups

Variable	Mean ± SD		t-value	p-value	Sig
	Control group N = 15	Aquatic therapy group N = 15			
Age (years)	23.80 ± 3.91	23.93 ± 3.26	− 0.101	0.920	NS
BMI (kg/m^2^)	22.373 ± 2.096	23.013 ± 1.996	− 0.856	0.399	NS

*SD = Standard deviation, *t-value = t-statistic, *P-value = probability, *Sig. = Significance, *NS = non-significant

The gender distribution between the Control and Aquatic therapy groups was assessed by the Chi-square test and revealed that there were no significant differences between groups (*p* = 0.465). Comparing the affected distribution for all patients in the Control and Aquatic therapy groups using the Chi-square test revealed that there were no significant differences between groups (*p* = 0.715) (Table 2).

**Table 2:** The frequency and the chi-squared test for comparison of gender and affected side distribution between the control and Aquatic therapy groups

Variable		*Group* A (Control)* N* = *15*	*Group B* (Aquatic therapy) *N* = *15*	χ2 value	*p*-value	Sig
Gender	Males	6 (40%)	7 (46.7%)	0.533	0.465	NS
	Females	9 (60%)	8 (53.3%)			
Affected side	Rt	8 (53.3%)	6 (40%)	0.133	0.715	NS
	Lt	7 (46.7%)	9 (60%)			

RT = Right, χ2 value = Chi-square statistic, P-value = probability, Sig. = Significance, LT = Left

Repeated measure MANOVA was conducted to study the effect of rehabilitation timing on pain intensity, Hop and Agility tests in both groups. There was a significant interaction effect of Aquatic therapy and time for VAS (*p* < 0.001), single hop (*p* < 0.001), triple hop (*p* < 0.001), cross-over hop (*p* < 0.001), IAT (*p* = 0.019) and ATT (*p* < 0.001). There was no significant interaction effect of Aquatic therapy and time for 6-MHT (*p* = 0.923). There was a significant main effect of time for all dependent variables (*p* < 0.001) except for 6-MHT (*p* = 0.939), and IAT (*p* = 0.099) (Table 3).

**Table 3:** Effect of timing of rehabilitation on all dependent variables

Repeated measure MANOVA			
Interaction effect (Group * time)			
VAS	F = 15.660		p < 0.001*
SHT	F = 15.730		p < 0.001*
THT	F = 14.766		p = 0.001*
CHT	F = 16.499		p < 0.001*
6-MHT	F = 0.009		p = 0.923
IAT	F = 6.159		p = 0.019*
ATT	F = 18.433		p < 0.001*
*Effect of time*			
VAS	F = 953.148		p < 0.001*
SHT	F = 36.040		p < 0.001*
THT	F = 22.224		p < 0.001*
CHT	F = 22. 682		p < 0.001*
6-MHT	F = 0.006		p = 0.939
IAT	F = 2.905		p = 0.099
ATT	F = 13.618		p = 0.001*

*VAS = Visual analoug scale, *SHT = single hop test, *THT = triple hop test, *CHT. = cross-over hop test, *6-MHT = 6-m hop test, IAT = Illinois agility test, *ATT = agility T test

*variable units: *VAS = cm, SHT = cm, THT = cm, CHT = cm, 6-MHT = cm, IAT = Secounds, ATT = Secounds

**Fig. 2:**
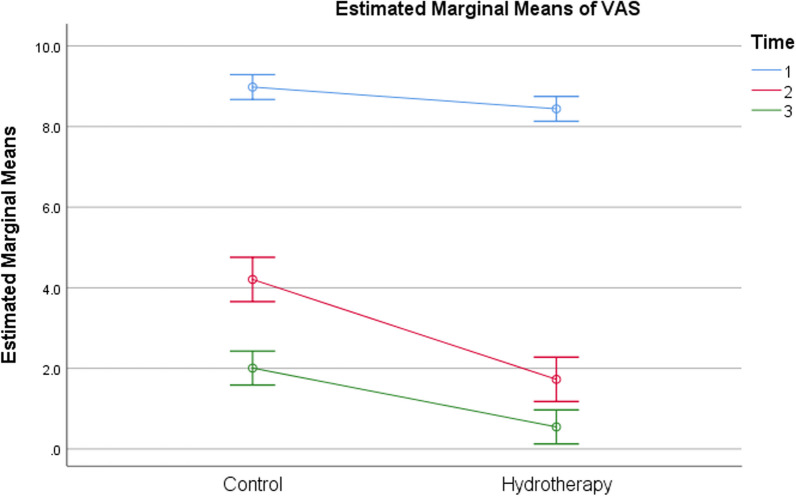
Estimated marginal mean values of pain intensity in both groups pre and post-week 4 and 6

**Fig. 3:**
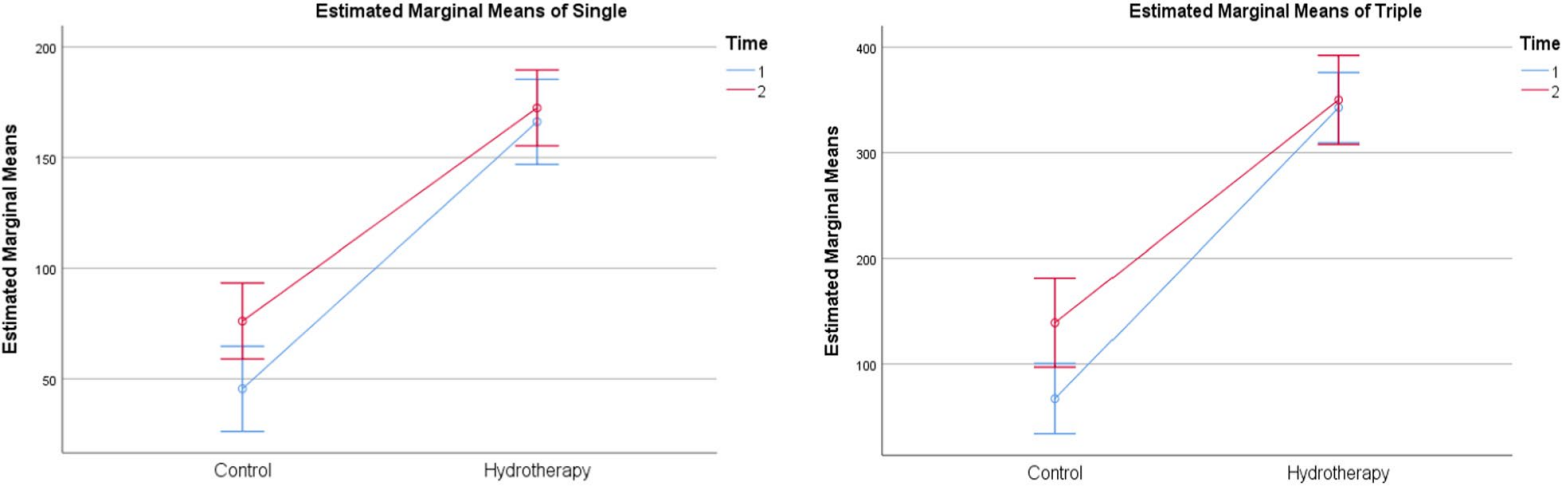
Estimated marginal mean values of single (left) and triple (right) leg hop test in both groups post rehabilitation at weeks 4 and 6

**Fig. 4:**
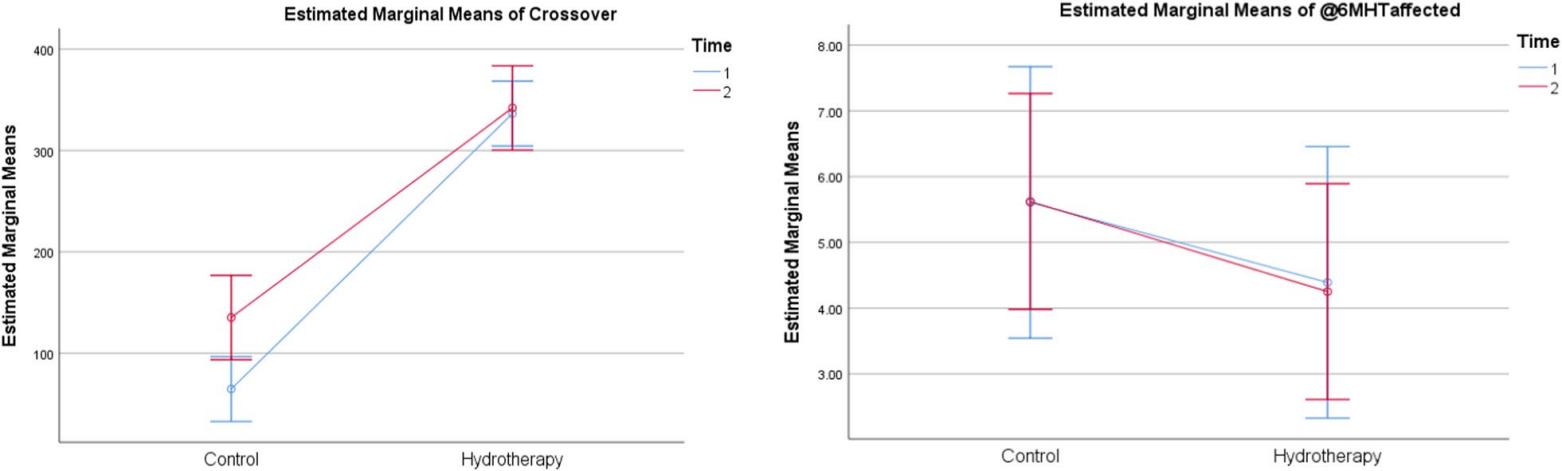
Estimated marginal mean of cross-over hop test (left) and 6-m hop test (right) in both groups post-rehabilitation at weeks 4 and 6

The effect of Aquatic therapy training on pain intensity levels showed a significant difference within and between-group comparisons (*p* < 0.001) (Fig. 2 and Table 4). The single-hop, the triple-hop test results were found significant within and between groups (*p* < 0.001) (Fig. 3 and Table 4). The cross-over hop test results showed significant within and between-group comparisons (*p* < 0.001) (Fig. 4 and Table 4). The 6-m hop test was the only variable that showed no significant within and between-group comparisons (*p* = 0.923) (*p* = 0.140) (Fig. 4 and Table 4).

**Table 4:** Mean, within and between-group comparisons in both groups

		Group A (Control) N = 15	Group B (Aquatic therapy) N = 15	Between-group comparison		
		$\overline{\rm X}$ ± SD	$\overline{\rm X}$ ± SD	MD (CI 95%)	*p*-value	Sig
VAS (cm)	Pre	8.98 ± 0.57	8.44 ± 0.60	− 0.540 (− .1039; − 0.9761)	0.017*	Sig
	Post W4	4.21 ± 1.32	1.73 ± 0.64	− 2.480 (− 1.702; − 3.258)	< 0.001*	Sig
	Post W6	2.01 ± 1.03	0.55 ± 0.46	− 1.460 (− 0.864; − 2.056)	< 0.001*	Sig
	MD pre Vs Post W6 (CI 95%)	− 6.973 (− 6.426; − 7.521)	− 7.893 (− 7.424; − 8.363)			
	*p*-value	< 0.001*	< 0.001*			
	Sig	Sig	Sig			
SHT (cm)	Post W4	45.47 ± 50.05	166.13 ± 12.17	120.667 (148.909;92.424)	< 0.001*	Sig
	Post W6	76.13 ± 44. 28	172.40 ± 12.21	96.267 (120.559;71.874)	< 0.001*	Sig
	MD (CI 95%)	30.667 (43.737; 17.596)	6.267 (8.073; 4.460)			
	*p*-value	< 0.001*	< 0.001*			
	Sig	Sig	Sig			
THT (cm)	Post W4	67.13 ± 88.41	342.73 ± 11.23	275.6 (322.73.228;8.467)	< 0.001*	Sig
	Post W6	139.13 ± 112. 22	350.07 ± 11.32	210.933 (270.585; 151.282)	< 0.001*	Sig
	MD (CI 95%)	72 (108.049; 35.95)	7.333 (9.130; 5.537)			
	*p*-value	0.001*	< 0.001*			
	Sig	Sig	Sig			
CHT (cm)	Post W4	64.60 ± 84.91	336.40 ± 12.38	271.8 (317.184;8.483)	< 0.001*	Sig
	Post W6	135.13 ± 110. 62	342 ± 11.37	206.867 (265.681;148.053)	< 0.001*	Sig
	MD (CI 95%)	70.533 (104.765; 36.30)	5.600 (7.527; 3.673)			
	*p*-value	0.001*	< 0.001*			
	Sig	Sig	Sig			
6-meter HT (seconds)	Post W4	5.61 ± 5.52	4.39 ± 0.11	− 1.215 (1.705; − 4.136)	0.401	NS
	Post W6	5.62 ± 4. 39	4.25 ± 0.15	− 1.371 (0.951; − 3.693)	0.237	NS
	MD (CI 95%)	0.016 (3.458; − 3.426)	− 0.140 (− 0.079; − 0.200)			
	*p*-value	0.992	< 0.001*			
	Sig	NS	Sig			
IAT (seconds)	Post W4	5.53 ± 14.75	18.55 ± 2.06	13.015 (− 4.858; 8.483)	0.002*	Sig
	Post W6	16.33 ± 16.48	16.54 ± 1.21	0.213 (20.891; 5.139)	0.960	NS
	MD (CI 95%)	10.797 (21.842; − 0.247)	− 2.005 (− 1.354; − 2.656)			
	*p*-value	0.055	< 0.001*			
	Sig	NS	Sig			
ATT (seconds)	Post W4	2.82 ± 7.46	10.89 ± 0.79	8.073 (12.042;4.103)	< 0.001*	Sig
	Post W6	15.91 ± 9.62	9.90 ± 0.73	− 6.013 (− 0.911;− 11.116)	0.023*	Sig
	MD (CI 95%)	13.097 (20.128; 6.065)	− 0.989 (− 0.715; − 1.264)			
	*p*-value	0.001*	< 0.001*			
	Sig	Sig	Sig			

$\overline{\rm X}$: Mean, SD: Standard deviation, MD: Mean difference, t value: Unpaired t value, *p*-value: Probability value, NS: Non-significant

**Fig. 5:**
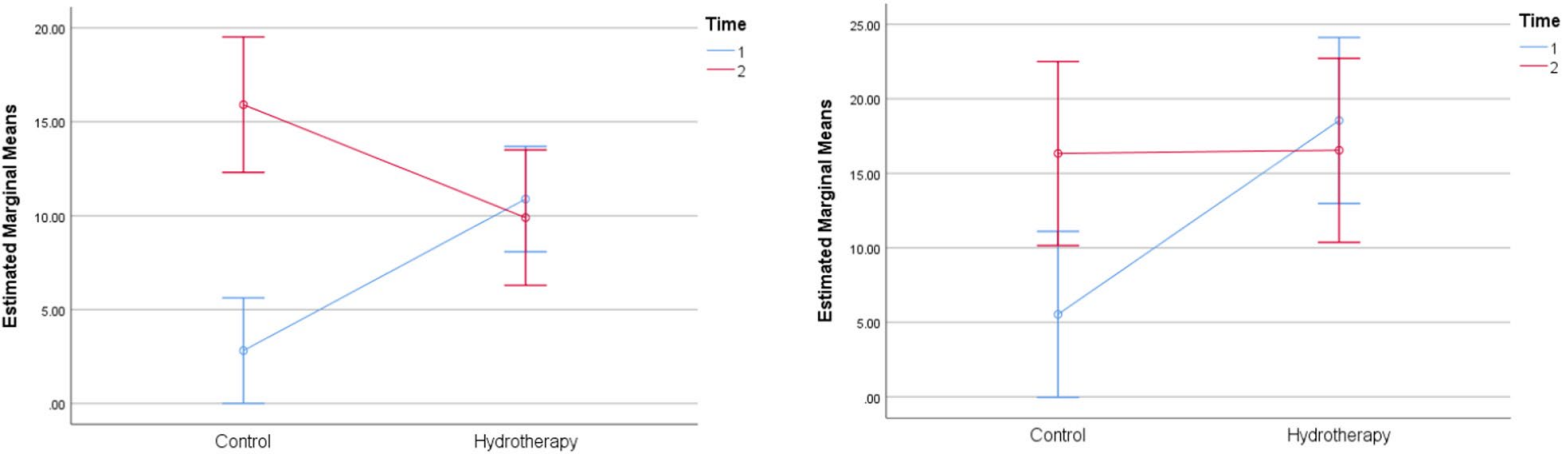
Estimated marginal mean of the agility T-test (left) and Illinois agility test (right) in both groups post-rehabilitation at weeks 4 and 6

The mean values of the agility T-test revealed significant within-group differences for the control (*p* = 0.001*) and aquatic therapy (*p* < 0.001*) groups. Furthermore, significant between-group comparisons for weeks 4 (*p* < 0.001*) and 6 (*p* = 0.023*) were observed (*p* = 0.001). The mean values of the Illinois agility test revealed significant within-group differences for the aquatic therapy group (*p* < 0.001*) while no significant difference was observed in the control group (*p* = 0.055). Significant between-group comparisons for week 4 (*p* = 0.002*) were observed while no significant between-group comparison for week 6 (*p* = 0.960) (Fig. 5 and Table 4).

For dynamic balance assessment, the mean value of the distance reached by the unsupported limb in the eight directions is shown in .

**Fig. 6:**
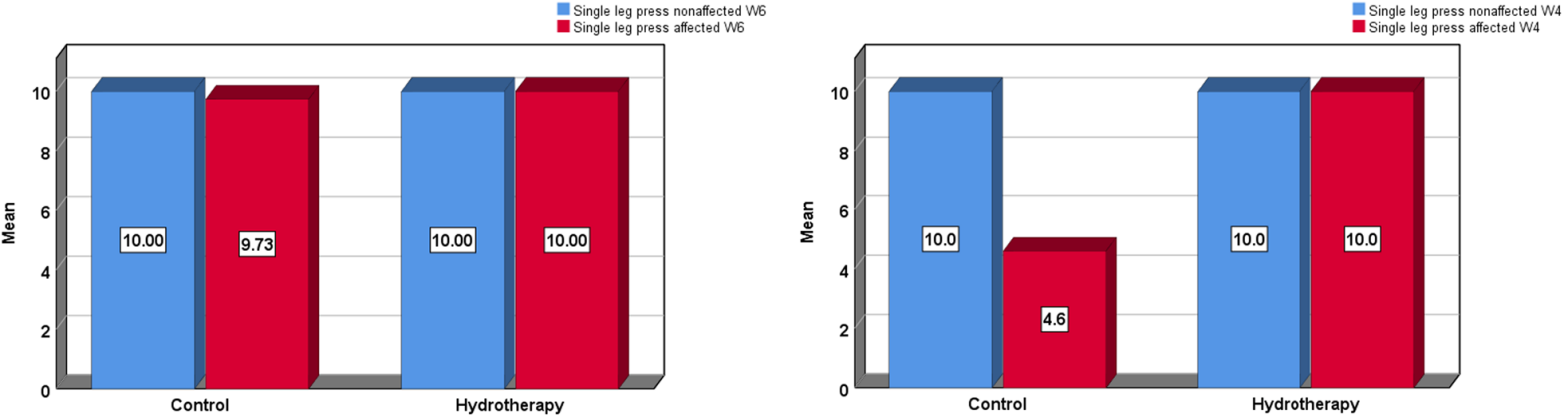
Mean values of the muscle power test of affected and non-affected at week 4(right) and week 6 (left) in both groups post-rehabilitation

**Fig. 7:**
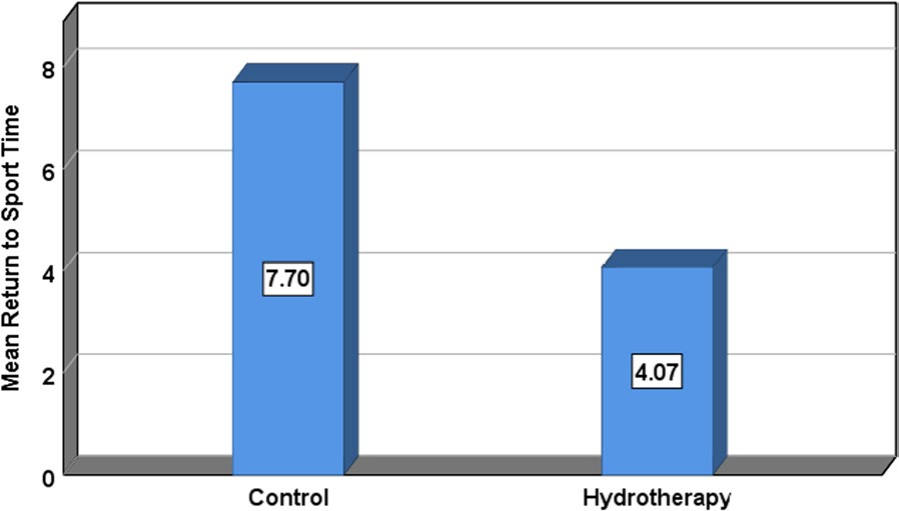
Mean values of return to sport time in both groups post-rehabilitation

Table 5. The Star excursion dynamic balance test (SEBT) revealed a significant difference between groups (*p* < 0.001*) as illustrated by a one-way analysis of variance ANOVA in Table 6. Finally, Muscle power testing by single leg press results showed a significant difference between groups (*p* < 0.001*) (Fig. 6). Lastly and most importantly the back-to-sport time revealed a significant difference in the return-to-sport time in favor of the Aquatic therapy group who returned faster than the control group (*p* < 0.001*) (Fig. 7).

**Table 5:** Descriptive statistics for dynamic balance assessment by SEBT

		N	Mean	SD	SE	95% Confidence Interval for Mean		Min	Max
						Lower bound	Upper bound		
*Descriptives*									
SEBT affected anterior	Group A	15	88.08	2.15	0.56	86.89	89.27	83.84	92.00
	Group B	15	93.21	3.05	0.79	91.52	94.90	84.85	97.87
	Total	30	90.65	3.68	0.67	89.27	92.02	83.84	97.87
SEBT affected anteromedial	Group A	15	85.92	2.67	0.69	84.44	87.39	80.81	90.59
	Group B	15	90.81	4.01	1.04	88.59	93.03	80.81	96.81
	Total	30	88.36	4.17	0.76	86.81	89.92	80.81	96.81
SEBT affected medial	Group A	15	92.56	2.51	0.65	91.17	93.95	86.87	95.56
	Group B	15	96.87	2.97	0.77	95.22	98.51	88.89	100.00
	Total	30	94.71	3.47	0.63	93.42	96.01	86.87	100.00
SEBT affected posteromedial	Group A	15	90.08	2.02	0.52	88.96	91.20	85.86	93.41
	Group B	15	94.21	3.95	1.02	92.03	96.40	84.85	97.89
	Total	30	92.15	3.73	0.68	90.75	93.54	84.85	97.89
SEBT affected posterior	Group A	15	94.02	2.83	0.73	92.45	95.59	88.57	98.89
	Group B	15	97.42	4.23	1.09	95.08	99.76	89.90	105.32
	Total	30	95.72	3.93	0.72	94.25	97.19	88.57	105.32
SEBT affected posterolateral	Group A	15	87.26	2.51	0.65	85.87	88.65	81.90	91.76
	Group B	15	93.98	4.36	1.13	91.56	96.40	82.83	102.13
	Total	30	90.62	4.89	0.89	88.79	92.45	81.90	102.13
SEBT affected lateral	Group A	15	83.23	3.17	0.82	81.47	84.98	76.53	87.06
	Group B	15	90.56	4.05	1.05	88.32	92.80	80.81	97.80
	Total	30	86.89	5.16	0.94	84.96	88.82	76.53	97.80
SEBT affected anterolateral	Group A	15	87.54	2.43	0.63	86.19	88.89	82.86	92.94
	Group B	15	90.95	3.98	1.03	88.75	93.15	80.81	97.80
	Total	30	89.24	3.68	0.67	87.87	90.62	80.81	97.80
SEBT affected average	Group A	15	88.59	2.09	0.54	87.43	89.74	85.10	91.76
	Group B	15	93.50	3.18	0.82	91.74	95.26	84.22	98.27
	Total	30	91.04	3.64	0.66	89.69	92.40	84.22	98.27

**Table 6:** ANOVA testing between groups for dynamic balance assessment by SEBT

		Sum of Squares	Mean Square	F	P-value	Sig
ANOVA						
SEBT affected anterior	Between Groups	197.307	197.307	28.36	< 0.001*	S
	Within Groups	194.803	6.957			
	Total	392.11				
SEBT affected anteromedial	Between Groups	179.506	179.506	15.487	< 0.001*	S
	Within Groups	324.538	11.591			
	Total	504.045				
SEBT affected medial	Between Groups	139.039	139.039	18.445	< 0.001*	S
	Within Groups	211.066	7.538			
	Total	350.105				
SEBT affected posteromedial	Between Groups	128.144	128.144	13.029	0.001	S
	Within Groups	275.383	9.835			
	Total	403.527				
SEBT affected posterior	Between Groups	86.941	86.941	6.728	0.015	S
	Within Groups	361.836	12.923			
	Total	448.777				
SEBT affected posterolateral	Between Groups	338.922	338.922	26.725	< 0.001*	S
	Within Groups	355.097	12.682			
	Total	694.019				
SEBT affected lateral	Between Groups	403.404	403.404	30.554	< 0.001*	S
	Within Groups	369.683	13.203			
	Total	773.086				
SEBT affected anterolateral	Between Groups	87.398	87.398	8.04	0.008	S
	Within Groups	304.354	10.87			
	Total	391.751				
SEBT affected average	Between Groups	181.208	181.208	25.011	< 0.001*	S
	Within Groups	202.861	7.245			
	Total	384.069				

*F value: F statistic, p-value: Probability value, S: significant (p < 0.05)

*SEBT* star excursion balance test

## Discussion

The findings of the present study demonstrate the superiority of an accelerated early intervention protocol utilizing a four-week Aquatic therapy training program for acute ankle sprain grade III in Elite professional athletes. A comparison with the traditional physiotherapy rehabilitation program revealed significant differences favoring the Aquatic therapy group in terms of ankle pain, overall functionality, dynamic balance, and various athletic performance and power measures. Notably, athletes following the suggested Aquatic therapy protocol achieved a faster return to sport, approximately 4.7 weeks after injury, compared to 7.7 weeks for land-based exercises used in the control group. These results challenge the common practice of immobilization and casting and recently rigid tapping as the initial treatment, favoring functional treatment and therapeutic exercises in enhancing athletic performance and reducing the risk of reinjury [1, 20].

The findings of this study are consistent with previous research highlighting the benefits of addressing severe acute ankle sprains in the sports population. Such injuries have a detrimental impact on players' performance, team morale, and competition-winning rates, in addition to imposing financial burdens and emotional distress on injured athletes [24].

Related research regarding this study showed no significant difference between aquatic therapy and land-based therapy regarding chronic ankle instability treatment [32]. Therefore, this study came as a highly needed one because it highlighted the benefit of aquatic training in the rehabilitation of acute ankle sprain grade 3 by showing very promising results.

Aquatic therapy presented strong historical beneficial usage and overall reduction of pain Together with both cardiovascular and musculoskeletal health beneficial effects through water buoyancy. However, it showed no significant difference from other types of therapy for some orthopaedic conditions such as osteoarthritis (OA) [17]. Therefore, these studies formed a strong base for the current study to support the superior results that were shown by this study.

Considering individual preferences and factors like cost, accessibility, and resource availability is crucial when selecting the most appropriate treatment for acute ankle sprains. Aquatic therapy may be particularly suitable for individuals who prefer water-based exercises or have limitations in weight-bearing activities, while traditional physical therapy may offer greater accessibility and cost-effectiveness for certain individuals.

The current study focused on the ultimate way to decrease the return to sport time while maintaining the optimal performance level, the least amount of pain, ideal balance and muscle power, which in return increased the general satisfaction and psychological welfare of both the athletes and their contractors. However, it was limited to the professional athletic population of mainly overhead athletes and high-contact sports that aged between 18 and 30 years and had recent injuries within 7 days.

The findings of the current study agreed with that of a previous one regarding edema and pain in specific musculoskeletal conditions of assuming the upright position during running in water. This upright posture resembles walking and non-weight-bearing exercise in warm water in the Aquatic therapy group [38]. Moreover, certain studies have indicated that Aquatic therapy could potentially alleviate pain in individuals with musculoskeletal injuries when compared to receiving no therapy. However, these studies did not find significant pain reduction benefits of Aquatic therapy when compared to ground exercises [28].

Previous research has consistently indicated that Aquatic therapy holds promise in enhancing performance outcomes among athletes with musculoskeletal injuries [1]. In line with these findings, this study demonstrated that Elite professional athletes participating in a four-week Aquatic therapy training program exhibited significant improvements in muscle power level, agility, and balance compared to those undergoing traditional physiotherapy rehabilitation. These results reinforce the notion that Aquatic therapy offers a valuable approach to optimizing athletic performance during the recovery process.

Several studies have examined the effects of Aquatic therapy on muscle power and performance parameters and have found supporting evidence for its efficacy[40]. Similarly, our study revealed significant improvements in muscle power measures among athletes who underwent the suggested Aquatic therapy protocol compared to those in the control group receiving conventional therapy. These consistent findings across studies underscore the impact of Aquatic therapy in enhancing muscle power levels, which is crucial for athletes' overall performance and competitive edge.

It is noteworthy that certain studies have explored the effects of Aquatic therapy on performance outcomes and have yielded consistent results with our findings [25]. For instance, investigations assessing the impact of Aquatic therapy on various athletic performance measures, such as speed, jumping ability, and power, have reported significant improvements following the intervention. Our study adds to this body of evidence, demonstrating that Elite professional athletes who underwent the Aquatic therapy protocol experienced superior performance outcomes, further substantiating the efficacy of Aquatic therapy in enhancing athletic performance.

Previous studies supported the findings of this study as they stated the benefits to the sports population because the issue of severe acute ankle sprains affects the performance of the players, the morale of the team and the competition's winning rate. Furthermore, it represents a financial burden on injured players in addition to their emotional comptonization [24].

Furthermore, individual preferences and factors such as cost, accessibility, and availability of resources were considered when selecting the appropriate treatment for acute ankle sprains. This study showed aquatic therapy as a more suitable method for individuals who prefer water-based exercises or have limitations in weight-bearing activities while maintaining the least cost-effective approaches available.

It is important to consider the limitations of this study. The sample size was relatively small, which might have affected the statistical power to detect subtle differences between the groups. Additionally, the duration of treatment and follow-up period in this study was limited to a specific timeframe, and the long-term effects of Aquatic therapy versus conventional physical therapy were not evaluated. Future studies with larger sample sizes and longer follow-up periods are needed to provide more comprehensive insights into the comparative effects of these treatment approaches.

While our study demonstrated significant positive outcomes of Aquatic therapy for grade III ankle sprains, several questions remain unanswered, presenting avenues for future research. Firstly, the long-term effects of aquatic therapy on functional outcomes, such as return to sports and prevention of recurrent sprains, need to be explored. Lastly, exploring the potential benefits of combining aquatic therapy with other complementary interventions, such as manual therapy or neuromuscular training, may further enhance the rehabilitation outcomes for individuals with grade III ankle sprains. Future research endeavors addressing these unanswered questions will contribute to a deeper understanding of the therapeutic potential of aquatic therapy and further optimize its application in clinical practice.

## Conclusion

Aquatic therapy is more effective than conventional physical therapy programs regarding early rehabilitation of acute ankle sprain grade III in Elite professional athletes for reducing pain intensity, improving dynamic balance and athletic performance and power and accelerating their return to sports time. Because aquatic therapy produces better outcomes, it is advised to be included in the rehabilitation programs of athletic patients with acute ankle sprains grade III.

## Data Availability

Any data will be available at the corresponding author upon request.
