# Correction: Effect of aquatic versus conventional physical therapy program on ankle sprain grade III in elite athletes: randomized controlled trial

**DOI:** 10.1186/s13018-026-06691-w

**Published:** 2026-02-18

**Authors:** Maryam M. Sadaak, Salwa Fadl AbdElMageed, Mona Mohamed Ibrahim

**Affiliations:** 1 Smouha Sporting Club, Alexandria, Egypt; 2 Department of Physical Therapy for Musculoskeletal Disorders and its Surgeries, Faculty of Physical Therapy, Cairo University, Cairo, Egypt

**Correction to: Journal of Orthopaedic Surgery and Research (2024) 19:400**

10.1186/s13018-024-04855-0

In Table 4 of this article [1], the mean value (X ± SD) of IAT (Illinois agility test) Post W4 and ATT (agility T test) Post W4 were incorrect and should have been 25.53 ± 14.75 and 22.82 ± 7.46 respectively.

Incorrect Table 4:

**Table 4:** Mean, within and between-group comparisons in both groups

		Group A (Control) N = 15	Group B (Aquatic therapy) N = 15	Between-group comparison		
		${\overline{\mathrm{X}}}$ ± SD	${\overline{\mathrm{X}}}$ ± SD	MD (CI 95%)	*p*-value	Sig
VAS (cm)	Pre	8.98 ± 0.57	8.44 ± 0.60	− 0.540 (− .1039; − 0.9761)	0.017*	Sig
	Post W4	4.21 ± 1.32	1.73 ± 0.64	− 2.480 (− 1.702; − 3.258)	< 0.001*	Sig
	Post W6	2.01 ± 1.03	0.55 ± 0.46	− 1.460 (− 0.864; − 2.056)	< 0.001*	Sig
	MD pre Vs Post W6 (CI 95%)	− 6.973 (− 6.426; − 7.521)	− 7.893 (− 7.424; − 8.363)			
	*p*-value	< 0.001*	< 0.001*			
	Sig	Sig	Sig			
SHT (cm)	Post W4	45.47 ± 50.05	166.13 ± 12.17	120.667 (148.909;92.424)	< 0.001*	Sig
	Post W6	76.13 ± 44.28	172.40 ± 12.21	96.267 (120.559;71.874)	< 0.001*	Sig
	MD (CI 95%)	30.667 (43.737;17.596)	6.267 (8.073; 4.460)			
	*p*-value	< 0.001*	< 0.001*			
	Sig	Sig	Sig			
THT(cm)	Post W4	67.13 ± 88.41	342.73 ± 11.23	275.6 (322.73.228;8.467)	< 0.001*	Sig
	Post W6	139.13 ± 112.22	350.07 ± 11.32	210.933 (270.585; 151.282)	< 0.001*	Sig
	MD (CI 95%)	72 (108.049; 35.95)	7.333 (9.130; 5.537)			
	*p*-value	0.001*	< 0.001*			
	Sig	Sig	Sig			
CHT (cm)	Post W4	64.60 ± 84.91	336.40 ± 12.38	271.8 (317.184;8.483)	< 0.001*	Sig
	Post W6	135.13 ± 110.62	342 ± 11.37	206.867 (265.681;148.053)	< 0.001*	Sig
	MD (CI 95%)	70.533 (104.765; 36.30)	5.600 (7.527; 3.673)			
	*p*-value	0.001*	< 0.001*			
	Sig	Sig	Sig			
6-meter HT (seconds)	Post W4	5.61 ± 5.52	4.39 ± 0.11	− 1.215 (1.705; − 4.136)	0.401	NS
	Post W6	5.62 ± 4.39	4.25 ± 0.15	− 1.371 (0.951; − 3.693)	0.237	NS
	MD (CI 95%)	0.016 (3.458; − 3.426)	− 0.140 (− 0.079; − 0.200)			
	*p*-value	0.992	< 0.001*			
	Sig	NS	Sig			
IAT (seconds)	Post W4	5.53 ± 14.75	18.55 ± 2.06	13.015 (− 4.858; 8.483)	0.002*	Sig
	Post W6	16.33 ± 16.48	16.54 ± 1.21	0.213 (20.891; 5.139)	0.960	NS
	MD (CI 95%)	10.797 (21.842; − 0.247)	− 2.005 (− 1.354; − 2.656)			
	*p*-value	0.055	< 0.001*			
	Sig	NS	Sig			
ATT (seconds)	Post W4	2.82 ± 7.46	10.89 ± 0.79	8.073 (12.042;4.103)	< 0.001*	Sig
	Post W6	15.91 ± 9.62	9.90 ± 0.73	− 6.013 (− 0.911; − 11.116)	0.023*	Sig
	MD (CI 95%)	13.097 (20.128; 6.065)	− 0.989 (− 0.715; − 1.264)			
	*p*-value	0.001*	< 0.001*			
	Sig	Sig	Sig			

${\overline{\mathrm{X}}}$: Mean, SD: Standard deviation, MD: Mean difference, t value: Unpaired t value, *p*-value: Probability value, NS: Non-significant

Correct Table 4:

**Table 4:** Mean, within and between-group comparisons in both groups

		Group A (Control) N = 15	Group B (Aquatic therapy) N = 15	Between-group comparison		
		${\overline{\mathrm{X}}}$ ± SD	${\overline{\mathrm{X}}}$ ± SD	MD (CI 95%)	*p*-value	Sig
VAS (cm)	Pre	8.98 ± 0.57	8.44 ± 0.60	− 0.540 (− .1039; − 0.9761)	0.017*	Sig
	Post W4	4.21 ± 1.32	1.73 ± 0.64	− 2.480 (− 1.702; − 3.258)	< 0.001*	Sig
	Post W6	2.01 ± 1.03	0.55 ± 0.46	− 1.460 (− 0.864; − 2.056)	< 0.001*	Sig
	MD pre Vs Post W6 (CI 95%)	− 6.973 (− 6.426; − 7.521)	− 7.893 (− 7.424; − 8.363)			
	*p*-value	< 0.001*	< 0.001*			
	Sig	Sig	Sig			
SHT (cm)	Post W4	45.47 ± 50.05	166.13 ± 12.17	120.667 (148.909;92.424)	< 0.001*	Sig
	Post W6	76.13 ± 44.28	172.40 ± 12.21	96.267 (120.559;71.874)	< 0.001*	Sig
	MD (CI 95%)	30.667 (43.737;17.596)	6.267 (8.073; 4.460)			
	*p*-value	< 0.001*	< 0.001*			
	Sig	Sig	Sig			
THT(cm)	Post W4	67.13 ± 88.41	342.73 ± 11.23	275.6 (322.73.228;8.467)	< 0.001*	Sig
	Post W6	139.13 ± 112.22	350.07 ± 11.32	210.933 (270.585; 151.282)	< 0.001*	Sig
	MD (CI 95%)	72 (108.049; 35.95)	7.333 (9.130; 5.537)			
	*p*-value	0.001*	< 0.001*			
	Sig	Sig	Sig			
CHT (cm)	Post W4	64.60 ± 84.91	336.40 ± 12.38	271.8 (317.184;8.483)	< 0.001*	Sig
	Post W6	135.13 ± 110.62	342 ± 11.37	206.867 (265.681;148.053)	< 0.001*	Sig
	MD (CI 95%)	70.533 (104.765; 36.30)	5.600 (7.527; 3.673)			
	*p*-value	0.001*	< 0.001*			
	Sig	Sig	Sig			
6-meter HT (seconds)	Post W4	5.61 ± 5.52	4.39 ± 0.11	− 1.215 (1.705; − 4.136)	0.401	NS
	Post W6	5.62 ± 4.39	4.25 ± 0.15	− 1.371 (0.951; − 3.693)	0.237	NS
	MD (CI 95%)	0.016 (3.458; − 3.426)	− 0.140 (− 0.079; − 0.200)			
	*p*-value	0.992	< 0.001*			
	Sig	NS	Sig			
IAT (seconds)	Post W4	25.53 ± 14.75	18.55 ± 2.06	13.015 (− 4.858; 8.483)	0.002*	Sig
	Post W6	16.33 ± 16.48	16.54 ± 1.21	0.213 (20.891; 5.139)	0.960	NS
	MD (CI 95%)	10.797 (21.842; − 0.247)	− 2.005 (− 1.354; − 2.656)			
	*p*-value	0.055	< 0.001*			
	Sig	NS	Sig			
ATT (seconds)	Post W4	22.82 ± 7.46	10.89 ± 0.79	8.073 (12.042;4.103)	< 0.001*	Sig
	Post W6	15.91 ± 9.62	9.90 ± 0.73	− 6.013 (− 0.911; − 11.116)	0.023*	Sig
	MD (CI 95%)	13.097 (20.128; 6.065)	− 0.989 (− 0.715; − 1.264)			
	*p*-value	0.001*	< 0.001*			
	Sig	Sig	Sig			

${\overline{\mathrm{X}}}$: Mean, SD: Standard deviation, MD: Mean difference, t value: Unpaired t value, *p*-value: Probability value, NS: Non-significant
